# The association between drinking water turbidity and gastrointestinal illness: a systematic review

**DOI:** 10.1186/1471-2458-7-256

**Published:** 2007-09-21

**Authors:** Andrea G Mann, Clarence C Tam, Craig D Higgins, Laura C Rodrigues

**Affiliations:** 1 Infectious Disease Epidemiology Unit, Department of Epidemiology & Population Health, London School of Hygiene & Tropical Medicine, Keppel Street, London, WC1E 7HT, UK

## Abstract

**Background:**

Studies suggest that routine variations in public drinking water turbidity may be associated with endemic gastrointestinal illness. We systematically reviewed the literature on this topic.

**Methods:**

We searched databases and websites for relevant studies in industrialized countries. Studies investigating the association between temporal variations in drinking water turbidity and incidence of acute gastrointestinal illness were assessed for quality. We reviewed good quality studies for evidence of an association between increased turbidity and gastrointestinal illness.

**Results:**

We found six relevant good quality studies. Of five studies investigating effluent water turbidity, two found no association. Two studies from Philadelphia reported increased paediatric and elderly hospital use on specific days after increased turbidity. A fifth study reported more telephone health service calls on specific days after peak turbidity. There were differences between studies affecting their comparability, including baseline turbidity and adjustment for seasonal confounders.

**Conclusion:**

It is likely that an association between turbidity and GI illness exists in some settings or over a certain range of turbidity. A pooled analysis of available data using standard methods would facilitate interpretation.

## Background

Microbial or chemical contamination of drinking water resulting from inadequate treatment at the plant or poor control of the distribution system can cause acute gastrointestinal illness [1-3]. Turbidity, a measure of the light refractiveness of water, is routinely used to indicate drinking water quality. Although microbiological contamination is commonly accompanied by increases in turbidity, other factors, including silt and organic matter, also affect turbidity levels of water leaving the treatment plant [4]. Limits of acceptable turbidity for water leaving the treatment plant vary between countries, but are generally below 1 or 2 nephelometric turbidity units (NTU) [5-8]; most effluent turbidity readings are well below these limits [9].

Outbreaks of gastrointestinal (GI) illness have been linked to incidents in which turbidity exceeded acceptable limits [10-16]. However, it is unclear whether endemic GI illness is associated with drinking water within acceptable turbidity levels ('normal' turbidity). In 1997, Schwartz *et al*. reported an association between variations in normal drinking water turbidity and endemic GI illness in children in Philadelphia [17]. The Environmental Protection Agency (EPA) concluded that the study's results were invalid, citing flaws in turbidity measurement and analysis techniques [18,19]. Despite this, subsequent studies in different settings have also suggested the existence of such an association [15,20-24].

We were commissioned by the Drinking Water Inspectorate of England and Wales to determine what evidence exists for an association between drinking water turbidity and endemic GI illness in settings with public water supplies similar to that in the United Kingdom (UK). We report the findings of a systematic critical review to assess the evidence for an association between turbidity levels of public drinking water supplies within acceptable quality limits and incidence of acute GI illness.

## Methods

We searched for all peer-reviewed papers published before December 2006 on the subject of water quality and diseases or poisonings with acute GI manifestations in PubMed, EMBASE, Aquatic Science and Fisheries Abstracts 3: Aquatic Pollution and Environmental Quality, and Industrial and Applied Microbiological Abstracts (Microbiology A) (see Appendix). We searched for non peer-reviewed papers using the System for Information on Grey Literature in Europe (SIGLE) and the following websites: Water Intelligence Online http://www.waterintelligenceonline.com/, Health Canada http://www.hc-sc.gc.ca/, Theses.com http://www.theses.com/, Theses Canada http://www.collectionscanada.ca/thesescanada/, and Proquest Digital Dissertations (for 2005 and 2006) http://wwwlib.umi.com/dissertations/gateway. We manually searched the reference lists of all papers that fulfilled our eligibility criteria and checked our results with experts in the field of drinking water quality.

Studies were included in the review if they investigated the effect of variations in turbidity of water from a treated public water supply (not a private well), either pre-treatment or effluent (post-treatment and leaving the treatment works), on the risk of acute GI illness in the population(s) served by that supply. We restricted the review to countries comparable to the UK in terms of water supply infrastructure and incidence of acute GI illness: Western Europe, the United States, Canada, Japan, Australia and New Zealand. Analyses comparing GI incidence before and after a treatment works upgrade were excluded, because system overhauls are one-time occurrences unrepresentative of the routine operation of the treatment plant. Similarly, outbreak investigations were excluded, since these events are unlikely to be representative of water quality generally. Intervention studies, where homes were fitted with active or sham drinking water filters at the tap, were excluded as they did not investigate the effect of turbidity specifically.

As exposure to drinking water in these settings is nearly universal, the association between turbidity and GI illness cannot easily be investigated using conventional epidemiological studies. All the studies identified used time-series approaches, in which the incidence of GI illness in a defined population is observed over time and compared at different levels of turbidity. Because such studies investigate the relationship between exposure and outcome over time, adequate adjustment must be made in the analysis for temporal patterns in exposure and outcome (and other relevant time-varying confounders such as temperature), in order to establish a causal rather than coincidental relationship.

Two reviewers (CCT, AGM) independently assessed each study according to a list of pre-determined criteria regarding appropriateness of the design and analysis, results and interpretation, and overall quality. The merits of each study, as compared with an optimum analysis, were discussed with two other reviewers (CDH, LCR) and summarized in text form. Criteria for an optimum analysis were as follows:

1. Exposure defined as daily turbidity at the treatment plant, expressed as the mean of several repeat measurements using standard equipment and units;

2. Outcome defined so as to minimize bias due to individuals' perception of exposure (e.g. hospitalizations for GI illness using International Classification of Diseases (ICD) codes);

3. Attempts made to minimize misclassification of exposure and outcome (e.g. using postcodes to match diagnoses to the catchment population of a treatment plant);

4. Use of appropriate analytical methods, taking account of seasonal variations in turbidity and acute GI illness, and other potential (time-varying) confounders.

Important differences were found between studies in terms of context and specification of analytical models. We thus considered it inappropriate to conduct a pooled analysis of study results.

## Results

**Figure 1:**
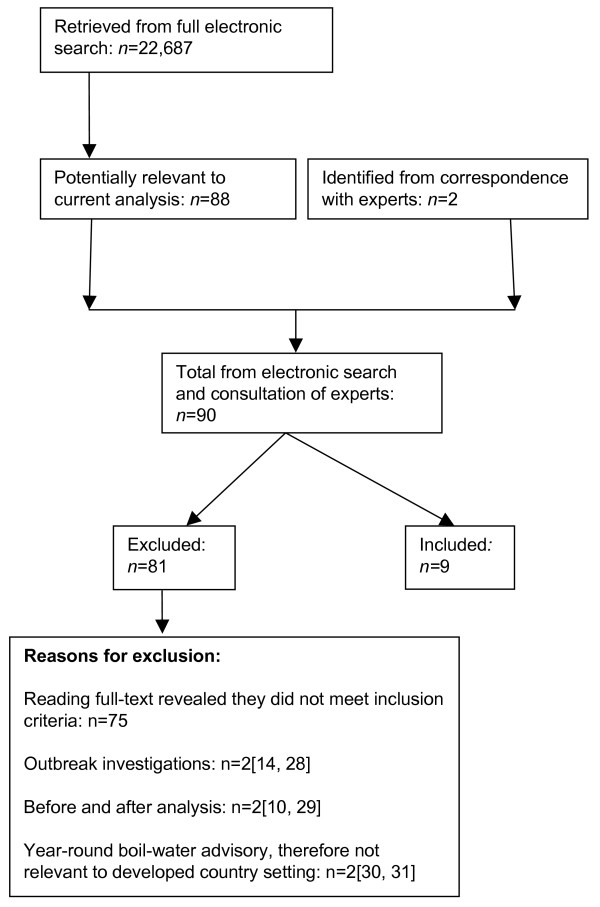
**Summary of studies retrieved, 1966–2006**.

Of 22,687 papers retrieved, we found two eligible studies investigating the association between GI illness and pre-treatment water turbidity [20,21], and eight studies of effluent water turbidity [15,17,21-26] (including Beaudeau *et al*. who studied both [21], [see Figure 1 and Additional File 1]).

### Studies of pre-treatment water turbidity

Of the two pre-treatment water studies identified, the study by Beaudeau *et al*. found no association and was excluded, because of problems with exposure definition and potential misclassification of both exposure and outcome [see Additional File 2][21].

The remaining pre-treatment study, by Aramini *et al*., defined exposure as the mean daily turbidity of water entering the treatment plant and the outcome as ICD-coded admissions, accident and emergency (A&E), and outpatient visits for acute GI illness, ascertained electronically[20]. Postcodes were used to match illness to treatment plant. Buffer zones, areas supplied by more than one treatment plant, were excluded to reduce exposure misclassification. Time-series regression using multivariable Poisson Generalised Additive Modelling (GAM) was used to allow for non-linear associations between turbidity and illness. Lags of up to 39 days for the effect of turbidity on GI illness were investigated (to encompass incubation periods of potential pathogens). An individual-level, logistic regression approach was also used to investigate temporal associations with turbidity among patients with GI illness compared with control patients with respiratory infections. Temporal exposure-response surface (TERS) plots were used to visualize associations. Such plots are three-dimensional surface graphs showing how the model-predicted risk of the outcome varies with increasing levels of the exposure at each lag; a flat or randomly varying TERS plot suggests no effect of turbidity on GI illness over the lags tested, while peaks on the plot indicate a consistent increase in incidence of GI illness over a specific range of turbidity at a specific time after that level of turbidity was recorded at the treatment plant. The authors took account of the most relevant confounders (long-term time trends, season, day-of-week, public holidays, temperature, and precipitation). Relative risks and odds ratios between 1.2 and 2.0 for both time-series and individual-level analyses were reported and several significant lags were highlighted (Table 1). The authors estimated that <2% of GI illness in areas of Vancouver served by the treatment plants studied is attributable to drinking water.

**Table 1:** Results reported in good quality studies

**Study**	**Effect measure (lag in brackets)**	**Over what unit of turbidity**	**Positive Lags (days)**	**Measure of population impact**
Schwartz, US (1997) [17]	Between 5–31% increase in A&E visits and admissions at various lags.	0.04 NTU	1, 4, 6–7†, 7–9†, 8, 10, 13	None stated.
Morris, US (1998) [23]	TERS plots suggest weak correlations; no statistical significance testing.	N/A	None.	N/A
Schwartz, US (2000) [24]	Between 5–15% (9–11) increase in admissions; 4–6 lag also significant for one plant; effect greater in those over 75 years (p < 0.0001)	0.035 NTU	4–6†, 9, 10, 11, 9–11†	None stated.
Aramini, Canada (2000) [20]	Relative rates and odds ratios between 1.2 and 2.0 for different watershed/age combinations.	*	3–6†, 6–9†, 12–16†, 21–29†	<2% GI illness estimated to be attributable to drinking water
Lim, Canada (2002) [25]	No significant measures of effect reported.	N/A	None.	N/A
Gilbert, Canada (2006) [22]	Relative rates of 1.33 (11), 1.53 (15) and 1.76 (17).	*	11, 15, 17	None stated.

*A relative rate and odds ratio of, for example, 1.2 represents a 20% increase in the likelihood of gastroenteritis associated with a particular level of turbidity at a particular lag, compared to the predicted probability associated with the mean turbidity effect for that particular lag, after adjusting for other parameters in the model

†combined

### Studies of effluent water turbidity

Of the eight eligible studies of effluent water turbidity, we excluded three (Morris 1996,[15] Beadeau, [21] and Naumova,[26] [see Additional File 2]). Problems with these three studies included: exposure defined as mean turbidity over more than one day (potentially masking daily effects), [15,21] non-specific outcome definition,[21] no geographic matching of outcome events to water supply,[15,21] and no adjustment for seasonal confounding effects [21,26].

Five of the eligible effluent water studies met our criteria for a good study [see Additional File 1] [17,22-25]. In all of these, exposure was defined as mean daily turbidity at the treatment plant. Four studies (Schwartz 1997,[17] Morris 1998,[23] Schwartz 2000,[24] and Lim[25]) used specific ICD codes for outcome definition, while Gilbert used the less-specific measure of electronic records of calls for certain GI symptoms to a telephone health line [see Additional File 2][22]. Postcodes were used to match diagnoses to treatment plant catchment areas in all five studies. Lim *et al*. also excluded buffer zones[25]. The studies used multivariable Poisson modelling, adjusting for seasonal variations in turbidity and acute GI illness, and other relevant potentially confounding factors (day-of-week effects, [17,22-25] long-term trends, [17,24,25] temperature, [17,24,25] and precipitation and public holidays [22,25]). The two studies by Schwartz assumed a linear relationship between turbidity and illness,[17,24] while the other studies allowed for non-linear associations.

The five studies reported varying results (Table 1). Lim *et al*. found no association [25]. Morris *et al*. (1998) did not report significance tests, but presented TERS plots showing no clear association between variations in turbidity and illness at any particular lag [23]. Gilbert *et al*. reported clear increases in calls for acute GI illness of between 33% and 76% at around 11, 15, and 17 days after high turbidity days, compared with the rate of calls the same number of days after a mean turbidity day [22]. The two Schwartz reports investigated the association of turbidity with paediatric and elderly GI illness, respectively, using the same turbidity data over the same time period. The authors estimated that a 0.04 NTU increase in turbidity on any given day resulted in up to a 31% increase in paediatric A&E visits and admissions four to 10 days later,[17] and up to a 15% increase in elderly hospitalizations nine to 11 days later [24].

## Discussion

We have conducted the most comprehensive review of the literature to date on the potential association between drinking water turbidity and incidence of endemic GI illness. We found nine studies conducted in settings relevant to the UK. Six of these were of sufficiently good quality to provide evidence of an association.

Aramini *et al*., one of the six studies meeting minimum quality criteria, investigated pre-treatment water turbidity [20]. Water was disinfected, but not filtered, before leaving the treatment works. The authors cited references regarding the decreased disinfection efficiency that may result from an increase in pre-treatment water turbidity (because of precipitation, for example). It is difficult to draw conclusions from this study, as the relationship between pre-treatment and effluent water, and the relevance of this as a measure of exposure, remains unclear; pre-treatment water turbidity may not be a good indicator of the quality of water leaving the plant.

Two of the five good-quality studies of effluent water turbidity (Lim [25] and Morris 1998 [23]) found no association, while the other three, two by Schwartz based on the same turbidity data, [17,24] and the Gilbert study,[22] found positive associations.

The Schwartz studies[17,24] were widely criticized by the EPA and others [18,19], who raised objections regarding the measurement of exposure and outcome, and the use of turbidity as an indicator of microbiological contamination. Inclusion of factors not associated with microbial content in the exposure would result in a 'noisy' proxy for exposure and dilute or mask any existing effect on GI illness. However, turbidity is commonly used as a crude measure of water quality and turbidity levels in breach of quality limits are often associated with outbreaks of GI illness [10-15]. If normal turbidity levels can act as a proxy for concentration of agents of GI illness, then this should be reflected in the lags at which associations with hospital admissions are found: chemicals and viruses should result in associations at shorter lags than bacteria or parasites, reflecting the relative incubation periods of these agents. Schwartz *et al*. reported two such clusters of lags at four to six and nine to 11 days, which they attributed to viral and parasitic agents respectively. Gilbert *et al*. reported significant lags (around 11, 15, and 17 days) at which increased rates of GI-related calls clustered. Unfortunately, it is difficult to demonstrate that certain lags are associated with specific agents, as GI-related ICD codes do not reliably differentiate between different GI pathogens and calls to a telephone health line are non-specific.

The EPA was also concerned that a large proportion of the turbidity readings were below the calibration limit of turbidity meters and that ICD codes potentially including non-infectious GI illness were included in the outcome definition. Although errors in turbidity measurement may be more common below the calibration limit, these are unlikely to be systematic and are reduced by using the mean of several daily measurements, while inclusion of GI events unrelated to exposure could dilute observed associations, but is unlikely to result in spurious associations being found where none truly exist [27].

A further issue in time-series studies is that, because associations are studied over multiple time lags between exposure and disease, some positive associations could be chance findings. Both the Schwartz and Gilbert studies addressed this by focusing on clusters of consecutive lags that predicted increases in GI hospitalizations, rather than the statistical significance of individual lags. If no association between turbidity and GI hospitalizations existed, positive and negative coefficients would be observed with an equal distribution across all lags tested, five percent of which would be significant at a conventional level of precision simply by chance. The probability of positive associations being observed over consecutive lags by chance is, however, much reduced [27], making the positive correlations observed by Schwartz and Gilbert unlikely to be chance associations.

### Differences between studies

Important differences were found between studies that may affect the interpretation and comparability of their results. Differences in the modelling strategy between studies, particularly the degree of seasonal adjustment employed, made studies difficult to compare. For example, the study by Lim adjusted for precipitation, whereas the Schwartz studies did not. However, it is unclear from the studies whether this variable is an important confounder. Gilbert did not include precipitation in the final model, after assessing goodness of fit of the model with and without precipitation, suggesting that it may not be an important confounder, at least in this setting. Adequate seasonal adjustment should account for potential confounding effects arising from the fact that precipitation is also seasonal. In addition, although rainfall may be biologically related to both exposure and outcome, at least part of its effect on GI illness will be through its effect on turbidity, and adjusting for it may not be of benefit unless other causal pathways exist.

Two other relevant questions are whether it is correct to assume a linear association between increases in turbidity and in GI illness (as done by Schwartz), and whether such an assumption holds throughout the range of turbidity and of incidence of GI illness. Indeed, mean turbidity in the Lim study was very much lower (0.06 NTU) than in the other studies (<0.3 NTU), but the range over which effluent turbidity varied was similar in all studies (about 0.1 to 0.5 NTU). A plausible explanation is that increases in turbidity levels around 0.06 NTU do not cause increases in illness, while increases from higher baseline turbidity levels do.

## Conclusion

Associations between drinking water turbidity and GI illness have been found in two settings, in people of various ages, but not in other settings. It is likely that studies observed different results because of differences in the mean turbidity level between settings. Important methodological differences, such as in the level of adjustment for seasonal confounders, might also help to explain conflicting results. An analysis of existing data using the same modelling approach, or a pooled analysis of the raw data from these and other settings, would facilitate a better understanding of currently available findings.

Further studies could consider alternative indicators of water quality; particle count data would improve the specificity of exposure definition, and could help differentiate between bacterial and parasitic agents based on their size, although it should be noted that non-pathogenic organisms are routinely found in water. Such technology is also more expensive and not widely available. Specificity of exposure definition could also be improved through the use of water quality data at different points in the distribution system, which would better represent the quality of water at the tap. Such data are, however, not easily available.

Finally, most studies did not estimate the proportion of GI illness in the population attributable to turbidity. This proportion depends on the magnitude of the turbidity effect at a given lag, the range over which turbidity has an effect, and the proportion of days for which turbidity is within this range (prevalence of exposure). Studies of drinking water are important because exposure is nearly universal, such that even small effects on GI illness could have considerable public health impact. Positive associations between drinking water quality and GI illness might suggest that threshold limits of acceptable turbidity (or, indeed, any other relevant measure of quality) should be reconsidered. However, any changes to drinking water regulations must be based not only on the demonstration of an association with GI illness, but also on the realistic impact that any regulatory changes would have on public health – in terms of the number of cases and societal costs averted – and the level of risk that is considered acceptable from drinking water.

## Competing interests

The author(s) declare that they have no competing interests.

## Authors' contributions

AGM conducted the literature search, summarized all eligible papers, participated in synthesis of findings and drafted the manuscript. CCT assisted with the literature search, summarized all eligible papers, participated in synthesis of findings and assisted in producing the manuscript. CDH and LCR participated in synthesis of findings. All authors read and approved the final manuscript.

## Appendix

### Search strategies

#### PubMed Search Strategy

##### Exposure

MeSH terms (separated by OR): Sanitation, Sewage,, Water Microbiology, Water Supply, Water Purification, Water Pollutants, Nephelometry and Turbidimetry, Water Pollution, Sanitary Engineering, Corrosion, Hazardous Substances

OR free text terms (separated by OR): water treat*, nephelomet*, turbid*, water source, water filt*, boil* water,, tap water, water discolo*, water qualit*, water deteriorat*, potable, corrosion by-product, corrosion by-product*, waste water, toxi*

##### Outcome

MeSH terms (separated by OR): Gastrointestinal Diseases, Cryptosporidiosis, Cryptosporidium, Giardiasis, Giardia, Bacterial Infections, Virus Diseases,, Poisoning

OR free text terms (separated by OR): diarr*, stomach, vomit*, waterborne, water-borne, water borne

##### Limit

Human

##### Restrict to

MeSH and related free-text terms (separated by OR): Canada, United States, Japan, Europe, Australasia

##### Exclude

Asia, Siberia, Georgia (Republic), South America, Central America, Africa, Mexico

#### EMBASE Search Strategy

##### Exposure

Exploded subject headings (separated by OR): Sewage treatment, Sewage, Microbiology, Water Supply, Water Management, Water Pollutant, Nephelometry, Water Pollution, Corrosion

OR Free text terms (separated by OR): water treat$, nephelomet$, turbid$, water source$, water filt$, boil$ water, tap water, water discolo$, water qualit$, water deteriorat$, potable, corrosion by-product, waste water

AND

##### Outcome

Exploded subject headings (separated by OR): Gastrointestinal Disease, Cryptosporidiosis,, Cryptosporidium, Cryptosporidium parvum, Giardiasis, Giardia, Giardia lamblia, Virus Infection, Bacterial infection Intoxication,

OR Free text terms (separated by OR): diarr$, stomach$, vomit$, waterborne, water-borne, water borne

##### Limit

Human

##### Restrict to

MeSH and related free-text terms (separated by OR): Canada, United States, Japan, Europe, Australia and New Zealand

##### Exclude

Asia, South Asia, Southeast Asia, South America, Central America, Africa, Mexico

#### Aquatic Science and Fisheries Abstracts 3: Aquatic Pollution and Environmental Quality and Industrial and Applied Microbiological Abstracts (Microbiology A) combined search strategy

Discolo* or turbid* or waterqualit* or drink*AND Gastrointestinal or diarr* (all free text terms)

## Pre-publication history

The pre-publication history for this paper can be accessed here:



## Supplementary Material

Additional file 1 Descriptive characteristics of all eligible studies. A description of each of the eligible studies in terms of setting, study population, length of study, and mean turbidity and gastrointestinal illness incidence in this setting. Click here for file

Additional file 2 Quality characteristics of all eligible studies. A summary of each eligible study with respect to exposure and outcome definition, measurement, and classification, the number of lags tested, the method of analysis, and the degree of confounder adjustment. Click here for file
